# Magnetoelectric Energy Harvesting for Industrial IoT Applications: Frequency-Tunable Converter with Enhanced Performance

**DOI:** 10.3390/s25216735

**Published:** 2025-11-04

**Authors:** Slim Naifar, Olfa Kanoun

**Affiliations:** 1 Laboratory of Electro-Mechanic Systems (LASEM), National School of Engineers of Sfax, University of Sfax, Sfax 3038, Tunisia; 2 Higher Institute of Applied Sciences and Technology of Gabès, University of Gabès, Gabès 6029, Tunisia; 3 Laboratory of Measurement and Sensor Technology (MST), Chemnitz University of Technology, 09126 Chemnitz, Germany; olfa.kanoun@etit.tu-chemnitz.de

**Keywords:** magnetoelectric energy harvesting, position-dependent magnetic forces, frequency tuning, industrial IoT, wireless sensors

## Abstract

The proliferation of wireless sensor networks in industrial Internet of Things (IIoT) applications demands sustainable power solutions that eliminate battery replacement requirements while maintaining operational reliability in varying vibration environments. This paper presents a frequency-tunable magnetoelectric (ME) energy harvester that addresses the fundamental challenge of frequency mismatch between ambient industrial vibrations and harvester resonance through position-dependent magnetic force manipulation. The proposed system employs a Terfenol-D/PMNT/Terfenol-D sandwich transducer mounted on a cantilever beam within an adjustable magnetic circuit, enabling continuous frequency tuning through air gap modification for different magnetic field configurations. A comprehensive theoretical framework incorporating position-dependent magnetic forces was developed to predict the system behavior. Additionally, Multi-walled carbon nanotube (MWCNT)-enhanced epoxy bonding layers with 2 wt.% concentration were analyzed and demonstrated six-fold power improvement over conventional epoxy. The experimental validation shows frequency tuning from 40 Hz to 65 Hz through air gap adjustment of only 1 mm, corresponds to a 62.5% tuning range. Further experimental investigation proves a ten-fold power output improvement up to 2 mW by employing a four-magnet circuit design compared to the two-magnet configuration through specific adjustment of the air gap width.

## 1. Introduction

Industrial Internet of Things (IIoT) represents a compelling challenge for both academic and industrial research communities. IIoT solutions are used for many applications including automation, security systems, supervision and predictive maintenance to avoid and predict system failure. Technological solutions related to IIoT rely mainly on massive diffusion of sensors, which require energy for their operations. Conventional energy supply solutions, i.e., batteries or wired connections, cannot meet the requirements of long-term operation and autonomy, which are decisive factors for the widespread installation of wireless sensors [1,2].

One of the most promising alternatives is harvesting energy from ambient sources that are otherwise wasted [3]. Powering wireless sensors by converting a part of their surrounding ambient energy can significantly revolutionize industrial environments in many application areas. Both installation and maintenance costs can be drastically reduced. This energy can take the form of vibration, temperature differences, light, or magnetic forces. The most promising source for harvesting energy for industrial applications is vibration due to its prevalence and the relatively high conversion efficiency of vibration converters [4].

Challenges associated with implementing energy harvesting techniques in general, and vibration energy transduction mechanisms specifically, are related to the suitability of these solutions for implementation in different industrial applications without failure. Considering existing vibration sources in industrial applications, they are mainly in forms of noisy signals having low amplitudes and including several characteristic frequencies [5]. On the other hand, vibration converters are characterized by a single resonance frequency and, therefore, have a relatively narrow operating frequency band. If the frequency of the excitation is not tuned to a vibration converter’s resonance, the energy harvesting system cannot perform its task effectively.

Numerous vibration harvesting converters have been presented in the literature, which rely mainly on four principles: electrostatic [6], piezoelectric [7,8], electromagnetic [9,10], and magnetoelectric [11], to convert vibration to electrical energy. Despite extensive research in this field, widening the operating frequency range of vibration converters remains a challenging task. Several mechanical tuning methods can be applied including: geometrical adjustment [12], straining the structure [13], generator arrays [14], and the use of negative springs [15]. However, applying these frequency tuning techniques often requires complex design implementations.

Frequency tunability has emerged as a critical design parameter for vibration energy harvesters deployed in real-world IoT applications where ambient vibration characteristics vary with operational conditions. Magnetic coupling approaches in hybrid harvester configurations have proven effective in creating multiple resonant frequencies and broadening operational bandwidth, with experimental demonstrations showing dual-peak frequency responses that capture energy across 4–5 Hz intervals centered at low-frequency ranges (8–12 Hz) [16]. For ultra low frequency applications below 10 Hz, magnetic force-based frequency tuning through adjustable magnet spacing has successfully optimized harvester performance, with hybrid systems employing frequency up-conversion mechanisms to broaden the working bandwidth of piezoelectric cantilever beams while combining piezoelectric and electromagnetic transduction to collect energy from the same excitation source [17]. Advanced spring-mass structures with carefully selected stiffness values (7.5 N/m) and proof masses (302 g) have been designed to achieve multi-modal vibrations within narrow frequency ranges characteristic of industrial applications such as freight train bogies, effectively dispersing the resonance bandwidth and minimizing frequency intervals between different vibrational modes to enhance energy harvesting efficiency across 9–12 Hz operational ranges [18]. One more interesting tuning technique relies on the use of magnetic force to alter the resonance frequency of the harvester. The tuning procedure consists of adjusting the distance between a magnet placed on the vibrating structure, e.g., a cantilever beam, and fixed magnets. Challa et al. [19] used this method for a piezoelectric micro-generator by applying a magnetic force perpendicularly to the cantilever. By varying the distance between fixed magnets and magnets placed at the free end of the cantilever, the resonance frequency could be tuned within the range from 22 Hz to 32 Hz while the untuned original value was 26 Hz.

Despite these advances in multi-modal bandwidth expansion and frequency tuning approaches, existing methods face several limitations. Autonomous tuning mechanisms, while effective, require complex control systems and additional power consumption for active adjustment [20]. Multi-modal hybrid harvesters achieve broadband operation through multiple discrete resonances rather than continuous frequency matching, limiting their effectiveness when ambient frequencies fall between resonant peaks. Mechanical spring-mass structures, though successful in dispersing resonance bandwidth, increase system complexity and device footprint.

**Figure 1:**
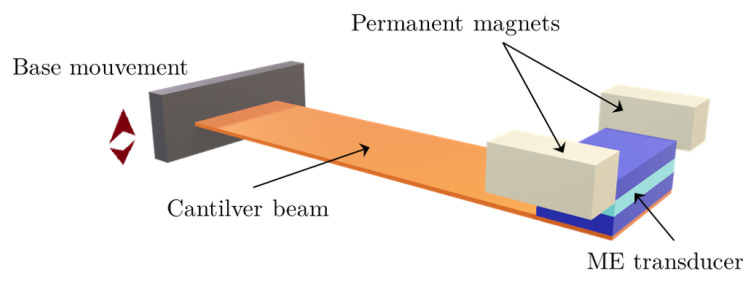
A schematic representation of the magnetoelectric energy harvester illustrating the cantilever beam with attached ME transducer, adjustable magnetic circuit with permanent magnets, and base excitation mechanism.

In magnetoelectric vibration converters, the magnetoelectric (ME) transducer is placed in the air gap of a magnetic circuit, and therefore a magnetic force is applied to the vibrating structure without the need for additional components (see Figure 1). However, most reported related studies have focused on optimizing the output performance of the converter through studying magnetic circuit arrangement, magnetic geometry, and ME transducer fabrication. Enhancing the frequency bandwidth of ME converters has been mainly addressed through multiple multilink ME transducers [21] and by varying excitation amplitude [22]. Despite the advantages of magnetoelectric energy converters, their practical deployment in industrial environments remains limited by the narrow operational bandwidth inherent to resonant systems. Recent studies have demonstrated that frequency deviations as small as 5–10% from resonance can reduce power output by an order of magnitude [23], making frequency adaptability crucial for real-world applications. Industrial machinery typically operates across a broad frequency spectrum, from 10–20 Hz in heavy rotating equipment to 50–120 Hz in motors and compressors [24], with these frequencies varying significantly due to load changes, equipment wear, and operational conditions.

Overall, most reported magnetoelectric energy harvesters have primarily focused on optimizing power output through the design of the magnetic circuit and the fabrication of the ME transducer [21,22], with frequency adaptability addressed mainly through passive multi-link configurations [21] or amplitude-dependent nonlinear effects [22], rather than through systematic position-dependent magnetic force manipulation.

This paper proposes a comprehensive approach to frequency tuning in magnetoelectric vibration converters by leveraging position-dependent magnetic forces and analyzing their effects on system equilibrium positions. The key innovation is the development of a theoretical framework that distinguishes between theoretical equilibrium positions and effective operating positions based on force-to-stiffness ratios. We investigated how different magnetic circuit configurations affect beam deflection and analyzed the bistability conditions that govern system behavior. The proposed method achieved frequency tuning from 40 to 65 Hz without the need for complex mechanical structures or additional components, making it particularly suitable for adaptive energy harvesting in industrial IoT applications.

The paper is structured as follows: Section 2 presents the harvester design, detailing the magnetoelectric converter architecture with the Terfenol-D/PMNT/Terfenol-D sandwich transducer, bonding layer, and adjustable magnetic circuit configurations. Section 3 develops the theoretical framework, establishing the governing equations with position-dependent magnetic forces, effective system stiffness analysis, and frequency tuning mechanisms. Section 4 provides finite element analysis, including characterization of Terfenol-D magnetic properties, magnetic field distribution, and force analysis for different configurations. Section 5 describes the implementation, results, and discussion, covering prototype fabrication with MWCNT-enhanced bonding layers and experimental investigations under both harmonic excitation and real vibration profiles. Finally, Section 6 concludes with key findings and their implications for industrial IoT applications.

## 2. Harvester Design

### 2.1. Magnetoelectric Converter Architecture

**Figure 2:**
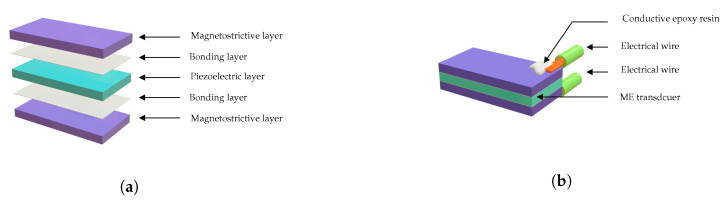
ME transducer assembly: (**a**) Exploded view of Terfenol-D/PMNT/Terfenol-D structure, (**b**) Assembled configuration with electrical connections.

The proposed magnetoelectric energy harvester integrates three essential components to achieve efficient vibration-to-electricity conversion with frequency tunability: a mechanical resonator in the form of a cantilever beam, a layered magnetoelectric transducer combining magnetostrictive and piezoelectric materials (see Figure 2), and an adjustable magnetic circuit providing both the bias field and tuning mechanism. The cantilever beam, fabricated from brass, provides the primary mechanical resonance structure with a calculated natural frequency of approximately 24 Hz when unloaded. The selection of brass ensures good mechanical properties, corrosion resistance, and ease of fabrication while maintaining consistent performance across the operational temperature range.

### 2.2. Bonding Layer

**Figure 3:**
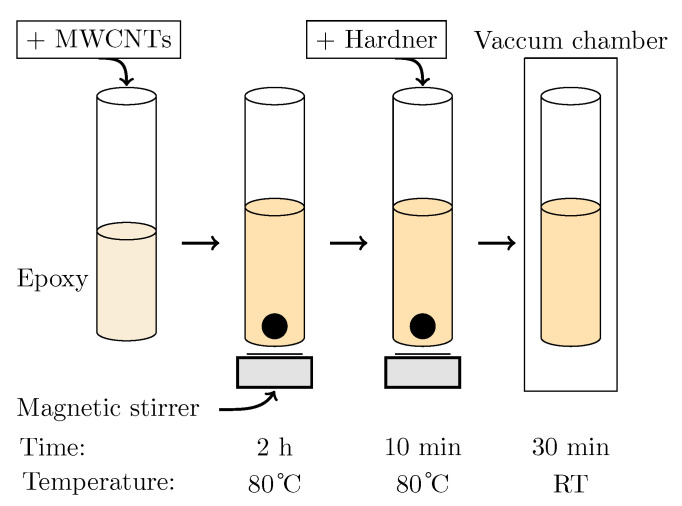
Preparation process of the polymer/MWCNTs for bonding the magnetostrictive and piezoelectric layers.

Three bonding layer formulations were investigated, including: conventional conductive epoxy, 1 wt.% MWCNT-enhanced epoxy, and 2 wt.% MWCNT-enhanced epoxy. The MWCNTs (6–9 nm in diameter, 5 μm length) were dispersed in epoxy resin by magnetic stirring at 600 rpm and 80 °C for 2 h, followed by addition of a hardener and vacuum degassing as shown in Figure 3.

### 2.3. Prototype Fabrication

**Figure 4:**
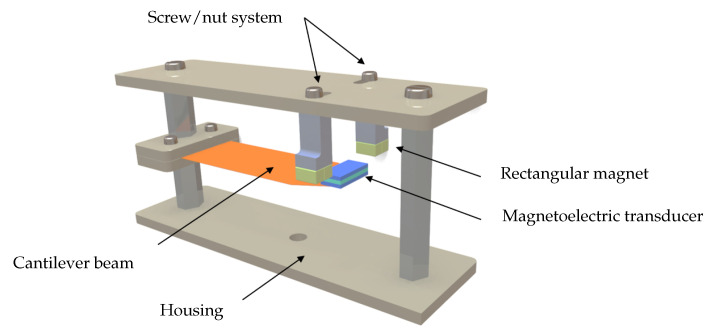
The structure of the magnetoelectric harvester showing the screw/nut adjustment mechanism, magnetic circuit housing, and cantilever mounting system.

The magnetoelectric (ME) energy harvester prototype consisted of a brass cantilever beam (50 mm × 20 mm × 0.2 mm) with the ME transducer attached at the free end. The magnetoelectric transducer employs a sandwich configuration consisting of one PMNT piezoelectric layer (12 mm × 6 mm × 1 mm) bonded between two Terfenol-D magnetostrictive layers of identical dimensions. This symmetric configuration maximizes strain transfer from the magnetostrictive layers to the piezoelectric core while maintaining mechanical balance. The Terfenol-D used in this study was supplied by ETREMA Products, Inc. (Ames, IA, USA), whereas the PMNT material was obtained from the Shanghai Institute of Ceramics, Chinese Academy of Sciences. The air gap width, denoted by e, represents the separation between the two halves of the magnetic circuit and can be finely adjusted through a screw–nut mechanism positioned between the magnet supports and the converter housing (see Figure 4). Adjusting this gap modifies the magnetic interaction between the permanent magnets and the two Terfenol-D layers, leading to variations in both the resonance frequency of the system and the output voltage generated by the ME transducer, due to the corresponding change in the magnetic field acting on the ME composite.

### 2.4. Magnetic Circuit Configuration

**Figure 5:**
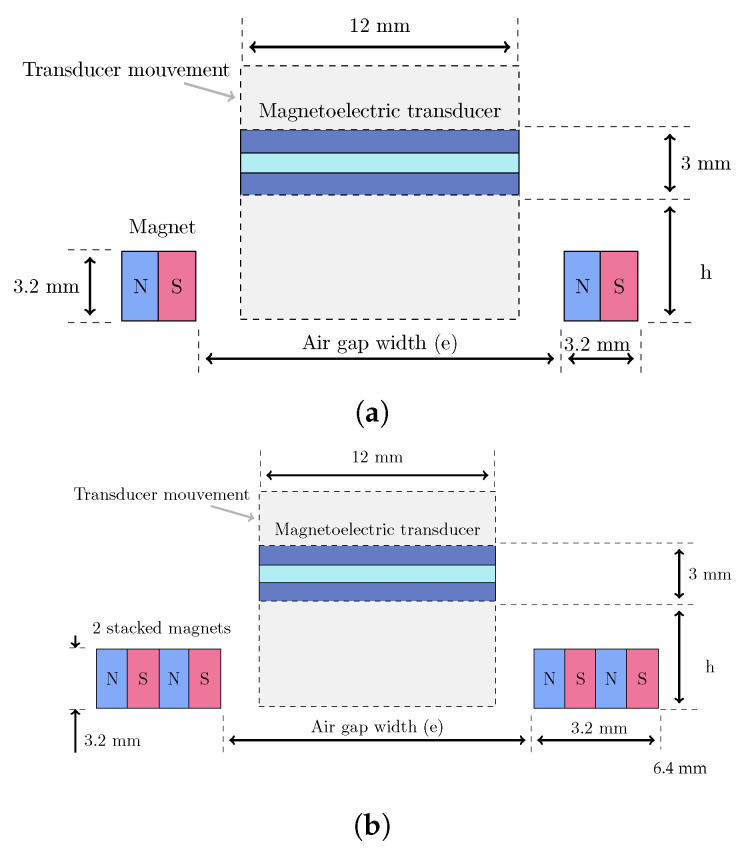
Magnetic circuit configurations: (**a**) two-magnet design with single magnets positioned symmetrically on both sides of the ME transducer and (**b**) four-magnet design with stacked magnets providing enhanced magnetic field strength. The air gap width e represents the adjustable parameter for frequency tuning.

The magnetic circuit serves dual purposes: providing the optimal bias magnetic field for ME transducer operation and enabling frequency tuning through magnetic force manipulation. Two configurations were investigated to understand the influence of magnetic field strength and distribution on harvester performance as shown in Figure 5. The magnetic circuit utilizes rectangular neodymium–iron–boron (NdFeB) permanent magnets with dimensions of 6.2 mm × 3.2 mm × 3.2 mm and a remanent flux density $B_{r}=1.32$ T.

The two-magnet configuration provides moderate magnetic fields suitable for investigating fundamental tuning behavior, while the four-magnet configuration generates stronger fields enabling extended tuning range and potential beam deflection effects. The air gap width e between the magnets can be precisely adjusted from 14 mm to 17 mm using a mechanical screw/nut mechanism, allowing fine control over the magnetic field strength and gradient experienced by the ME transducer.

## 3. Theoretical Framework

### 3.1. Governing Equation

**Figure 6:**
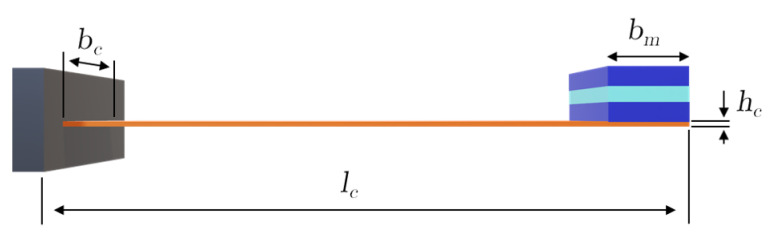
Lateral view of the cantilever beam showing geometric parameters length $l_{c}$, width $b_{c}$, thickness $h_{c}$, and ME transducer with mass $m_{t}$ at the tip.

The dynamic behavior of the frequency-tunable magnetoelectric energy harvester can be described by a single-degree-of-freedom system incorporating position-dependent magnetic coupling effects. The equation of motion for the cantilever tip displacement z(t) relative to the base excitation is given by the following:

(1) $$M_{eff}\ddot{z}+c\dot{z}+\left[k_{beam}+k_{ME}\left(H\right)\right]z+F_{mag}\left(z,H\right)=M_{eff}a_{b}\sin\left(\Omega t\right)$$

where $M_{eff}$ is the effective mass consisting of the ME transducer mass plus an equivalent mass contribution from the cantilever beam, *c* is the linear damping coefficient, $k_{beam}$ is the cantilever beam stiffness, $k_{ME}\left(H\right)$ represents the field-dependent stiffness contribution from magnetostrictive material properties, $F_{mag}\left(z,H\right)$ represents the position-dependent magnetic force, $a_{b}$ is the base acceleration amplitude, and Ω is the excitation angular frequency. The cantilever beam stiffness for a rectangular cross-section is the following:

(2) $$k_{beam}=\frac{3E_{c}J_{c}}{l_{c}^{3}}$$

where $E_{c}=100$ GPa is the Young’s modulus of brass, $J_{c}=b_{c}h_{c}^{3}/12$ is the second moment of area with $b_{c}=20$ mm width and $h_{c}=0.2$ mm thickness, and $l_{c}=50$ mm is the cantilever length (see Figure 6).

### 3.2. Position-Dependent Magnetic Force Analysis

The magnetic force acting on the ME transducer in the non-uniform magnetic field generated by the permanent magnets significantly influences system dynamics and enables frequency tuning through air gap adjustment. The position-dependent magnetic force can be derived from the gradient of magnetic energy. Following the approach presented by Blyakhman et al. [25], the general form of this force is the following:

(3) $$F=\frac{\mu_{0}\chi V}{2(1+N\chi )}\times \frac{\partial H^{2}\left(z\right)}{\partial z}$$

where *N* represents the demagnetization factor of the magnetostrictive material. For Terfenol-D layers in our ME transducer with negligible demagnetization effects (N≈0) in the operating field range and using the relationship $\chi =\mu_{r}-1$, the position-dependent magnetic force can be expressed as the following:

(4) $$F_{mag}\left(z\right)=-\frac{\partial U_{mag}}{\partial z}=-\frac{\mu_{0}\left(\mu_{r}-1\right)V_{Terfenol}}{2}\frac{\partial H^{2}\left(z\right)}{\partial z}$$

where $\mu_{0}=4\pi \times 10^{-7}$ H/m is the permeability of free space, $\mu_{r}$ is the relative permeability of Terfenol-D, $V_{Terfenol}$ is the volume of the magnetostrictive material, and H(z) is the position-dependent magnetic field strength obtained from the finite element analysis.

### 3.3. Effective System Stiffness and Frequency Tuning

The total effective stiffness of the system becomes position and field dependent as follows:

(5) $$k_{eff}\left(z,H\right)=k_{beam}+k_{mag}\left(z\right)+k_{ME}\left(H\right)$$

where $k_{mag}\left(z\right)=-\partial F_{mag}/\partial z$ represents the magnetic stiffness dependent on position and $k_{ME}\left(H\right)$ accounts for the field-dependent properties of the material. For practical implementation, and following the approach presented in [26], the resonance frequency of the system can be expressed as the following:

(6) $$f_{res}=\frac{1}{2\pi }\sqrt{\frac{k_{eff}\left(z_{op},H\right)}{M_{eff}}}=f_{0}\sqrt{1+\frac{k_{mag}\left(z_{op}\right)+k_{ME}\left(H\right)}{k_{beam}}}$$

where $f_{0}=\left(1/2\pi \right)\sqrt{k_{beam}/M_{eff}}$ is the natural frequency without magnetic effects and $z_{op}$ is the effective operating position. The effective operating position represents the displacement of the ME transducer’s center plane (middle plane) relative to the geometric center of the magnetic circuit, where z=0 corresponds to the position where the middle plane of the ME converter coincides with the middle plane of the magnets. For enhanced precision in frequency predictions, $z_{op}$ is determined experimentally using laser displacement measurements, as this approach accounts for practical factors including the additional stiffness contribution from electrical connection wires that can significantly affect the cantilever’s effective stiffness and consequently influence the equilibrium operating position.

This formulation demonstrates that frequency tuning is achieved through adjustment of magnetic force gradients through air gap variation, which directly affects $k_{mag}\left(z_{op}\right)$.

### 3.4. Field-Dependent Material Properties

The magnetostrictive Terfenol-D material exhibits field-dependent elastic properties known as the effect ∆E [27], as the following:

(7) $$E_{Terfenol}\left(H\right)=E_{0}\left[1+\alpha_{E}\tanh\left(H/H_{0}\right)\right]$$

where $\alpha_{E}=1.5$ represents the maximum relative change and $H_{0}$ is the characteristic field strength. This field-dependent stiffness contribution is incorporated into the system model through $k_{ME}\left(H\right)$ in Equation (5).

## 4. Finite Element Analysis

### 4.1. Terfenol-D Magnetic Property Characterization

Accurate finite element modeling of the ME harvester requires precise characterization of the Terfenol-D’s nonlinear B–H relationship. The magnetic behavior of the Terfenol-D exhibits strong nonlinearity and hysteresis, particularly near saturation. To address this issue, we employed the B–H Curve Checker application in COMSOL Multiphysics version 6.1 to optimize the Terfenol-D magnetic properties. This application implements the Simultaneous Exponential Extrapolation (SEE) method with specific modifications to ensure physical precision and numerical stability [28,29]. The SEE method describes the B–H curve near and in the saturation region using the analytical relation as follows:

(8) $$B\left(H\right)=B_{s}\left(1-ae^{-bH}\right)+\mu_{0}H$$

where $B_{s}$ is the saturation induction, *a* and *b* are coefficients determined from the measured data, and $\mu_{0}$ is the vacuum permeability. This equation ensures a physically realistic asymptotic behavior at high field strengths.

The differential relative permeability, which is critical for numerical convergence in nonlinear problems, can be derived as the following:

(9) $$\mu_{r,D}-1=\frac{B_{s}ab}{\mu_{0}}e^{-bH}.$$

In our implementation, the coefficients *b* and $B_{s}a$ were calculated using boundary data points near saturation. Additionally, the application applied linear interpolation as a low-pass filter to eliminate non-physical oscillations in the differential permeability curve, ensuring numerical stability while preserving the essential magnetic characteristics of the Terfenol-D.

**Figure 7:**
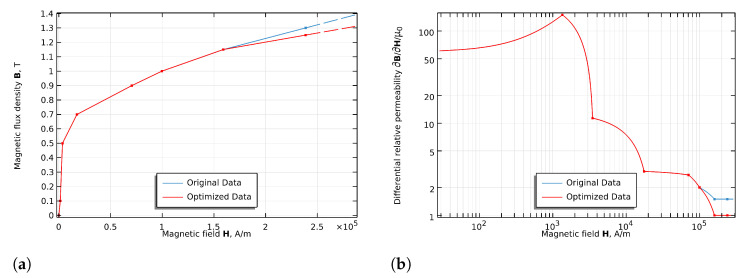
Comparison between original and optimized magnetic properties of the Terfenol-D: (**a**) B–H curves showing magnetic flux density versus magnetic field intensity and (**b**) differential relative permeability curves highlighting the smoothing effect of the optimization process.

Figure 7 presents a comparison between the original experimental data for the Terfenol-D and the optimized magnetic model.

As shown in Figure 7a, both the original and optimized B–H curves follow similar trajectories in the measured region, but the optimized curve provides a physically accurate extrapolation in the high-field region where experimental data are unavailable. More significantly, Figure 7b demonstrates the smoothing effect on the differential permeability curve, eliminating potentially problematic oscillations while maintaining the essential magnetic behavior.

The optimization process preserved the key magnetic characteristics of the Terfenol-D at lower field strengths while providing a more physically consistent behavior at higher field intensities. The original data points (0 to 238732.5 A/m for *H* and 0 to 1.3 T for *B*) were refined to produce a smooth curve that approaches magnetic saturation in a physically realistic manner.

The optimized B–H curve maintains accuracy within 2% of experimental data in the operational range (0–200 kA/m) while providing smooth extrapolation to saturation. The differential permeability curve shows the elimination of measurement noise that would otherwise cause convergence issues in nonlinear simulations.

### 4.2. Finite Element Model and Configuration Design

**Figure 8:**
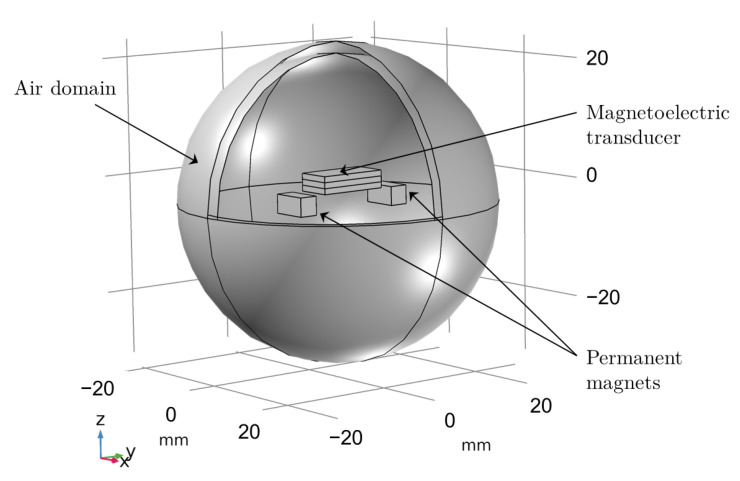
Finite element model showing the ME transducer positioned in the magnetic field created by the permanent magnet configuration.

Finite element simulations were performed to determine the magnetic field distribution and position-dependent characteristics for different configurations. The simulation incorporated nonlinear material properties and complex three-dimensional field distributions. The finite element model is presented in Figure 8. A summary of the material properties used to describe the Terfenol-D, and PMNT are shown in Table 1.

To systematically investigate the magnetic force effects and frequency tuning mechanisms, finite element simulations were conducted for three specific configurations among those tested experimentally as the following:

- Effect of magnet number: To study the influence of magnet quantity on system performance, two configurations with fixed air gap (e = 15 mm) were compared: two-magnet and four-magnet designs.
- Effect of air gap variation: To investigate air gap influence on magnetic forces, the four-magnet configuration was analyzed with reduced air gap from 15 mm to 14 mm.

### 4.3. Magnetic Field Distribution and Force Analysis

**Figure 9:**
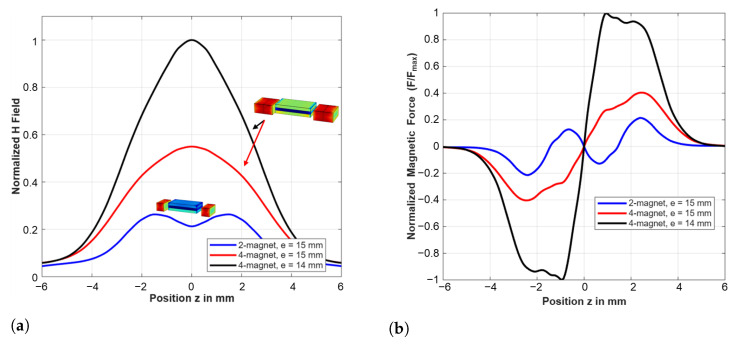
Finite element analysis results for three magnetic configurations: (**a**) normalized magnetic field distribution showing field strength variations, with the four-magnet, e = 14 mm configuration (black) providing highest field strength, and (**b**) normalized position-dependent magnetic force.

The finite element analysis reveals distinct magnetic field characteristics for each configuration, as shown in Figure 9. The four-magnet design with reduced air gap (e = 14 mm) provides the highest field strength, reaching normalized values near unity, while the two-magnet configuration exhibits lower but more uniform field distribution across the ME transducer region.

The magnetic force characteristics show highly nonlinear position dependence, with the four-magnet configuration at reduced air gap exhibiting the strongest force gradients. These steep gradients create significant magnetic stiffness contributions that dominate the system’s dynamic behavior. Figure 9b shows that the ME transducer experiences strongly nonlinear force gradients $\partial F_{mag}/\partial z$ for above 2 mm variation of the ME position. The magnitude of the resulting amplitude-dependent stiffness $|k_{mag}\left(z\right)|$ decreases as displacement increases, creating softening-type nonlinearity. This mechanism explains the amplitude jump shown in the results reported in Section 5.3. The ME converter’s operating position $z_{op}$ was determined experimentally. The corresponding magnetic stiffness $|k_{mag}\left(z_{op}\right)|=|\partial F_{mag}/\partial z{|}_{z_{op}}|$ was extracted from results shown in Figure 9 and used in Equation (6) for resonance frequency prediction.

## 5. Experimental Characterization and Performance Evaluation

### 5.1. Experimental Setup

**Figure 10:**
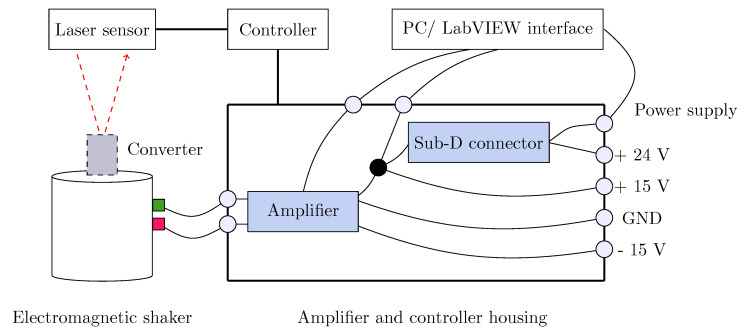
A schematicof the experimental setup showing the electromagnetic shaker.

The experimental setup is shown in Figure 10. The external vibrations were generated by an electrodynamic shaker (VebRobotron Type 11077) driven by a LabVIEW routine through a closed-loop controller. The housing of the converter was screwed to the shaker. A measurement card NI PCI 6259 was used as a signal source. A triangulation sensor (OptoNCDT ILD 1800) with 1 μm resolution and 10 mm measurement range was used to measure the time-varying displacement of the converter. In order to control the shaker and to adjust the output current of the measurement card, an amplifier was employed. Furthermore, two power supplies were used for both the laser sensor and the shaker controller. The open-circuit and load voltages were measured by a digital oscilloscope (LeCroy WaveRunner 6050A).

### 5.2. MWCNT-Enhanced Bonding Layer

**Figure 11:**
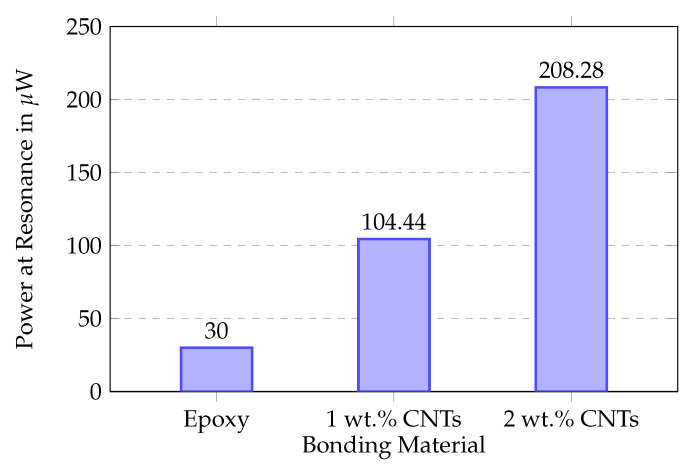
Power output comparison for different bonding materials. The 2 wt.% MWCNT composite achieves 210 μW peak power (e = 16 mm).

The optimization results demonstrate that MWCNT concentration significantly affects power output performance. The 2 wt.% MWCNT formulation achieved optimal performance with substantial power output improvements across the frequency range, reaching a peak of about 210 μW at 45 Hz as shown in Figure 11. This six-fold enhancement over conventional epoxy results from improved electrical conductivity and mechanical properties of the bonding interface, enabling more efficient strain transfer between the Terfenol-D magnetostrictive layers and the PMNT piezoelectric core. The 1 wt.% MWCNT concentration provided intermediate performance improvements, while higher concentrations beyond 2 wt.% showed diminishing returns due to agglomeration effects that compromise the uniform dispersion of nanotubes within the epoxy matrix. Therefore, all subsequent experiments employed the optimized 2 wt.% MWCNT bonding formulation to ensure maximum energy conversion efficiency.

Beyond power enhancement, MWCNT-enhanced bonding layers may also improve the long-term stability of the device under industrial operating conditions. Previous studies on MWCNT-reinforced epoxy adhesives have shown that the incorporation of MWCNTs significantly reduces shear strength degradation compared with neat epoxy after two weeks of hygrothermal aging (100% RH, 60 °C) [34], indicating possibility of enhanced durability and environmental resistance of the bonding layer used in this work.

### 5.3. Experimental Performance Validation

**Figure 12:**
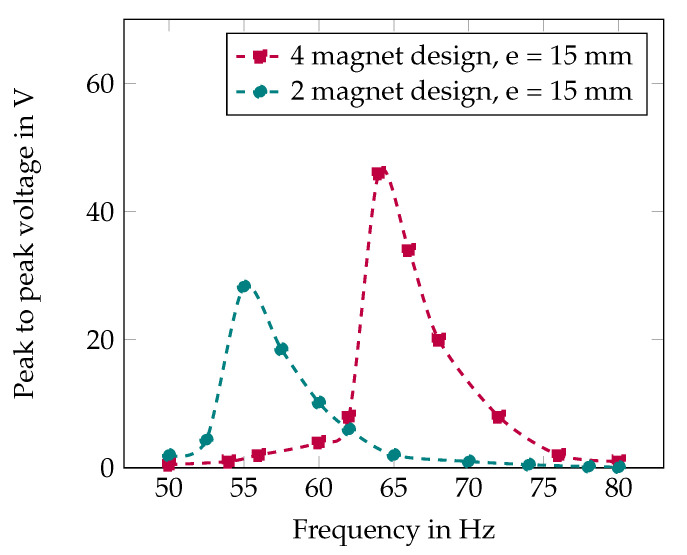
Frequency response for two-magnet and four-magnet design at different air gaps e = 15 mm.

**Figure 13:**
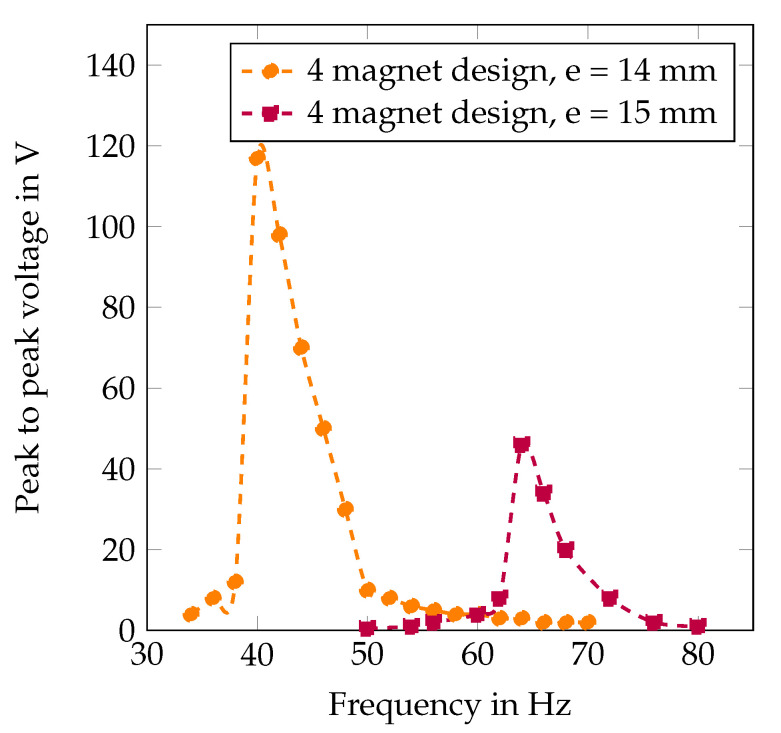
Maximum frequency tuning capability with four-magnet configuration showing the effect of air gap reduction.

The experimental characterization across three configurations: two-magnet (e = 15 mm), four-magnet (e = 15 mm), and four-magnet (e = 14 mm) is shown in Figure 12 and Figure 13.

The two-magnet configuration (e = 15 mm) achieves resonance at 55 Hz with 28 V peak voltage. Transitioning to the four-magnet configuration at the same air gap shifts resonance to 65 Hz with 46 V peak voltage—a 64% voltage enhancement resulting from increased magnetic flux density (0.45T versus 0.28T). The four-magnet configuration with reduced air gap (e = 14 mm) exhibits bistable behavior, operating at $z_{op}$ = 3.8 mm and achieving the lowest resonance frequency of 40 Hz with 120 V peak-to-peak voltage. This configuration delivers approximately 2 mW peak power, representing a ten-fold improvement over the two-magnet baseline.

The complete frequency tuning range spans 40–65 Hz (62.5% bandwidth) through 1 mm air gap adjustment combined with magnetic configuration changes. Voltage output remains above 60 V peak-to-peak across the entire tuning range, ensuring consistent power availability for wireless sensor applications. It is worth noting that, measurement repeatability tests, involving complete dismounting and remounting of the harvester on the shaker between trials, demonstrated variations within 6% of the reported values, confirming the robustness and statistical reliability of the claimed performance improvements.

### 5.4. Theoretical Model Validation

Table 2 demonstrates excellent agreement between theoretical predictions and experimental measurements, with errors below 5% for all configurations. The analytical framework accurately captures frequency shifts induced by magnetic stiffness variations, including the distinct behavior of the four-magnet (e = 14 mm). The model incorporates the nonlinear B–H curve of the Terfenol-D (Figure 7), accounting for magnetic saturation effects where $\mu_{r}$ decreases with increasing field strength. Field-dependent properties of material (∆E effect) contribute less than 2% to total stiffness, while damping effects induce frequency shifts below 0.2% for typical damping ratios (ζ<0.05), justifying their exclusion from the model. Experimental repeatability within ±1 Hz across multiple measurement cycles validates both the magnetic field simulation and force calculation methodology despite parameter uncertainties inherent in material properties and geometric tolerances.

### 5.5. Response Under Real Vibration Profiles

To validate practical applicability, the frequency-tunable ME converter was tested using two MATLAB R2024b-generated vibration profiles designed to emulate ambient industrial vibrations: (1) a broadband random signal with frequency content distributed across 20–80 Hz and peak spectral density around 45 Hz and (2) a second random signal with distributed frequency content in the 40–65 Hz range. Both profiles were band-limited to 80 Hz due to shaker system constraints.

Two converter configurations were evaluated: two-magnet (e = 16 mm, resonance: 45 Hz) and four-magnet (e = 15 mm, resonance: 65 Hz), selected to demonstrate frequency-selective energy harvesting from broadband excitation.

**Figure 14:**
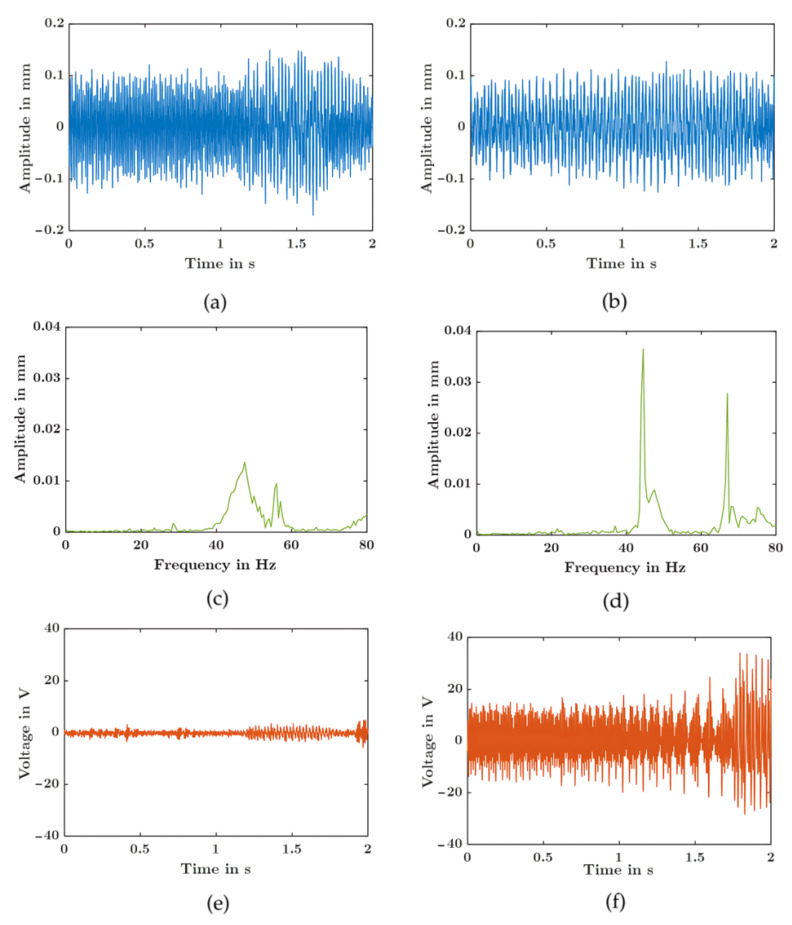
Response under real vibration profiles: (Left column, **a**,**c**,**e**): two-magnet configuration (e = 16 mm)—(**a**) applied vibration profile, (**c**) FFT spectrum of input signal, and (**e**) voltage output versus time; and (Right column **b**,**d**,**f**): four-magnet configuration (e = 15 mm)—(**b**) applied vibration profile, (**d**) FFT spectrum of input signal, and (**f**) voltage output versus time.

Figure 14 shows the test results. Subplots (a,b) display the applied vibration profiles, (e,f) present the FFT spectra revealing the characteristic frequencies in the input signals, and (c,d) show the voltage output versus time for the two-magnet configuration (e = 16 mm, resonance at 45 Hz) and four-magnet configuration (e = 15 mm, resonance at 65 Hz), respectively.

Under the complex multi-frequency excitation, the four-magnet configuration produced significantly higher voltage amplitude than the two-magnet design due to better alignment with dominant frequency components. By deploying configurations tuned to complementary resonances (45 Hz and 65 Hz), the system effectively harvested energy across the broader frequency spectrum, demonstrating the practical value of frequency tuning for real-world vibrations where characteristics vary with operating conditions.

It is important to note that ME converters exhibit a threshold behavior due to the magnetic attraction between the transducer and permanent magnets. While magnetic forces do not create physical contact, they can prevent oscillation until vibration amplitude exceeds a minimum threshold. This is evident in Figure 14e, where despite the presence of frequency content around 45 Hz in the spectrum (Figure 14c), the voltage output remains minimal because the excitation amplitude at resonance is insufficient to overcome the magnetic forces. In contrast, the four-magnet configuration (Figure 14f) achieves strong voltage output due to better frequency matching with the 65 Hz component, demonstrating that effective energy harvesting requires both frequency alignment and sufficient excitation amplitude. This threshold characteristic underscores the critical importance of precise frequency tuning for reliable harvester operation in real-world applications.

## 6. Conclusions

This work presents a comprehensive framework for frequency-tunable magnetoelectric energy harvesters addressing the critical challenge of frequency mismatch in industrial IoT applications. The integration of position-dependent magnetic force analysis with experimental validation demonstrates that systematic frequency tuning from 40 to 65 Hz can be achieved through minimal air gap adjustment (1 mm), representing a 62.5% operational bandwidth.

The key technical contributions include: (i) development and validation of a theoretical model incorporating position-dependent magnetic forces with <5% prediction error across all configurations; (ii) demonstration of ten-fold power enhancement (up to 2 mW) through optimized four-magnet configuration compared to baseline two-magnet design; and (iii) implementation of 2 wt.% MWCNT-enhanced bonding layers achieving six-fold power improvement over conventional epoxy at resonance.

The experimental validation under both harmonic and broadband excitation profiles confirms the harvester’s capability to adapt to varying industrial vibration environments.

The ability to maintain sufficient output across the entire tuning range ensures consistent power availability for wireless sensor nodes. The demonstrated performance characteristics directly address real-world industrial IoT deployment requirements. The 40–65 Hz tuning range covers typical machinery frequencies from heavy rotating equipment to motors and compressors, the 2 mW power output is sufficient for commercial wireless sensor nodes, and the simple mechanical tuning mechanism enables field adjustment without additional power consumption. These characteristics position this technology as a practical solution for battery-free operation in predictive maintenance, structural health monitoring, and process automation applications where vibration characteristics vary with operational conditions. Future research should focus on autonomous tuning mechanisms to eliminate manual adjustment, integrated power management, and multi-modal energy harvesting to enhance reliability and power density across diverse operating conditions.

## Figures and Tables

**Table 1:** Material properties used in finite element simulation.

Material	Property	Value	Unit	References
Terfenol-D	Density	9250	kg/m^3^	[30,31]
	Young’s modulus	30	GPa	
	Poisson’s ratio	0.5	–	
	Magnetostriction coeff.	1600	ppm	
	Relative permittivity	1	–	
PMNT	Density	8100	kg/m^3^	[32,33]
	Young’s modulus	20	GPa	
	Poisson’s ratio	0.32	–	

**Table 2:** Validation of theoretical frequency predictions.

Configuration	Air Gap	$z_{\mathrm{op}}$	$f_{\mathrm{res}}$ (Theory)	$f_{\mathrm{res}}$ (Exp.)	Error
	**(mm)**	**(mm)**	**(Hz)**	**(Hz)**	**(%)**
two-magnet	15	2.38	52.21	55 ±1	−5.1
four-magnet	15	2.44	62.41	64 ±1	−2.5
four-magnet	14	0.93	41.31	40 ±1	+3.3

## Data Availability

The data presented in this study are available on request from the corresponding author.
